# Association between prior gestational diabetes mellitus and adverse pregnancy outcomes

**DOI:** 10.3389/fendo.2026.1826255

**Published:** 2026-04-22

**Authors:** Mengqing Weng, Haibing Gao, Julan Peng, Huayou Li, Lili Chen, Han Yang, Jie Wang

**Affiliations:** 1 Medical Records Library, People’s Hospital of Longhua, Shenzhen, China; 2 People’s Hospital of Longhua, Shenzhen, China; 3 Department of Obstetrics, People’s Hospital of Longhua, Shenzhen, China; 4 Information Technology Department, People’s Hospital of Longhua, Shenzhen, China

**Keywords:** adverse pregnancy outcomes, gestational hypertension, postpartum anemia, prior gestational diabetes mellitus, subsequent pregnancy

## Abstract

**Objective:**

This study aims to examine the independent association between a history of gestational diabetes mellitus (GDM) and adverse maternal-fetal outcomes in subsequent pregnancies, with the objective of informing evidence-based clinical management strategies for this high-risk population.

**Methods:**

This retrospective study included women who delivered at Shenzhen Longhua District People’s Hospital between 2015 and 2025. Participants were categorized into a PGDM group (n=332) and a non-PGDM group (n=994) based on prior history of GDM. Propensity score matching was employed to control for confounding factors, and logistic regression analysis was used to assess the association between PGDM and adverse pregnancy outcomes.

**Results:**

Pregnant women with prior GDM had significantly higher risks of cesarean delivery (32.8% vs. 26.9%), GDM recurrence (50.0% vs. 11.8%), and gestational hypertension (3.0% vs. 1.2%) (all p<0.05). Multivariate analysis indicated that a history of PGDM independently predicted GDM recurrence (OR = 7.320, 95%CI: 5.473–9.791), gestational hypertension(OR = 2.924, 95% CI: 1.154–7.405), and reduced gestational age (OR = 0.88 per week, 95% CI: 0.792–0.981), while decreasing the risk ofPostpartum Anemia (OR = 0.694, 95% CI: 0.488–0.986). Neonatal outcomes showed no differences between groups.

**Conclusion:**

A history of PGDM significantly increases the risk of GDM recurrence and gestational hypertension in subsequent pregnancies, and is associated with earlier delivery gestational age. Notably, a history of PGDM is also associated with a reduced risk of postpartum anemia.

## 1 Background

Gestational diabetes mellitus (GDM), a prevalent metabolic disorder in pregnancy, is rising globally, paralleling the increasing prevalence of obesity and type 2 diabetes (1). Data from the International Diabetes Federation (IDF) indicate that the global prevalence of GDM is approximately14.0%, positioning it as a major public health concern with significant implications for maternal and neonatal health (2). This condition is closely associated with multiple adverse pregnancy outcomes, such as preterm birth, neonatal intensive care unit admission, and preeclampsia (3–6). However, the impact of GDM extends beyond the affected pregnancy. Accumulating evidence indicates that GDM is an independent risk factor for future type 2 diabetes and cardiovascular disease in women, underscoring the long-term health implications of gestational metabolic disturbance (3). In recent years, research has further focused on the “legacy effect” or “metabolic memory” of GDM, suggesting it may shape a unique risk profile for subsequent pregnancies (7). This risk profile, potentially underpinned by mechanisms such as persistent insulin resistance, impaired β-cell function, and chronic subclinical inflammation (8), places women with prior gestational diabetes mellitus(PGDM) at elevated risk for adverse outcomes in later pregnancies—even in the absence of prepregnancy hyperglycemia.

Currently, research on the association between PGDM and outcomes in subsequent pregnancies remains limited by insufficient evidence and inconsistent conclusions. Existing studies consistently demonstrate that PGDM is a strong predictor of GDM recurrence (with recurrence rates ranging from 30% to 84%) (7, 9),but its independent impact on other key maternal and fetal outcomes remains a subject of debate. Some studies have reported associations between PGDM and adverse outcomes in subsequent pregnancies, including macrosomia (10), gestational hypertensive disorders (11), and increased cesarean section rates (12, 13), while others have failed to confirm these associations after controlling for confounders such as prepregnancy BMI, interpregnancy interval, and gestational weight gain (14). Therefore, this study performed a retrospective cohort analysis to systematically assess the independent association between PGDM and adverse maternal-fetal outcomes in subsequent pregnancies, aiming to provide more precise evidence-based guidance for the management of subsequent pregnancies in women with PGDM.

## 2 Materials and methods

### 2.1 Study ethics and design

Data for this retrospective cohort study were obtained from the electronic medical record database of Longhua District People’s Hospital, Shenzhen City, Guangdong Province, China, and were collected from January 1, 2015, to June 30, 2025. The extracted data contained maternal demographic characteristics, medical and obstetric history, and information on maternal and infant outcomes. The study followed the principles of the Declaration of Helsinki and was approved by the Medical Ethics Committee of Longhua People’s Hospital. Given the retrospective nature of the study, the Medical Ethics Committee of Longhua People’s Hospital waived the requirement for informed consent. (Longhua People’s Hospital Ethical Review [2026] No. (019)).

### 2.2 Definitions

Gestational diabetes mellitus is diagnosed at 24–28 weeks of gestation or at the first obstetric examination after 28 weeks of gestation, using the American Diabetes Association (ADA) criteria, and the diagnosis is confirmed by meeting any of the following thresholds: fasting glucose ≥5.1 mmol/L, 1-hour glucose ≥10.0 mmol/L after oral dextrose administration, or 2-hour glucose ≥8.5 mmol/L (15). Infants were classified based on their birth weight percentiles for the same gestational age: those between the 10th and 90th percentiles were defined as appropriate for gestational age (AGA), those below the 10th percentile as small for gestational age (SGA), and those above the 90th percentile as large for gestational age (LGA) (16). Postpartum Anemia: Hemoglobin (Hb) <110 g/L (17).

### 2.3 Patient inclusion and study groups

**Figure 1:**
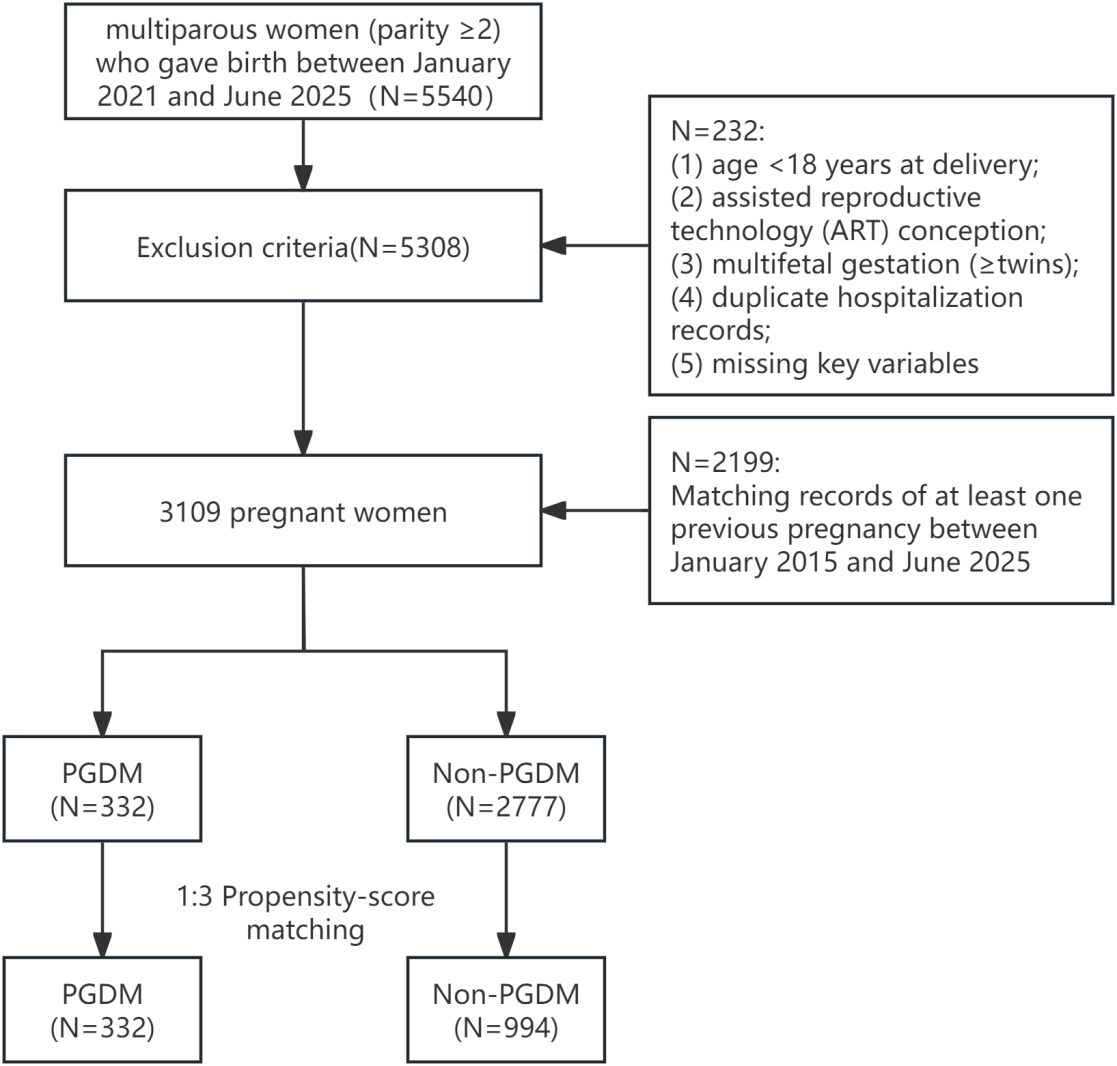
Data exclusion criteria.

This retrospective cohort study included multiparous women (parity ≥2) who delivered at Longhua District People’s Hospital, Shenzhen, China between January 2021 and June 2025, with at least one documented prior pregnancy in the same hospital from January 2015 to June 2025. All analyzed pregnancies met the criteria of ≥20 weeks’ gestation with complete antenatal records (including demographic characteristics, laboratory tests, and delivery outcomes). Exclusion criteria were: (1) maternal age <18 years at delivery; (2) assisted reproductive technology conception; (3) multifetal gestation (≥twins); (4) duplicate hospitalization records; and missing key variables (including oral glucose tolerance test results, birth weight, or maternal complication data), as shown in **Figure 1**.

### 2.4 Data collection

To ensure the reliability and validation of the database, data were entered by the first author and independently reviewed by the second and third authors. The following information was extracted from the electronic database: baseline maternal characteristics(maternal age, days of hospitalization, ethnicity, occupation, Interpregnancy interval in months, blood type, number of pregnancies, number of delivery, history of miscarriage); neonatal outcomes (neonatal sex, birth weight, NICU, gestational age); and maternal adverse pregnancy outcomes (Cesarean section, Gestational diabetes, premature, Premature Rupture of Membranes, Eclampsia, Gestational hypertensive disorders, Meconium-Stained Amniotic Fluid, Gestational Anemia, Postpartum Anemia, Postpartum Hemorrhage, Antepartum Hemorrhage, Hypothyroidism in Pregnancy, Hyperthyroidism in Pregnancy, Infection of the amniotic sac and fetal membranes, Pregnancy with obesity).

### 2.5 Statistical analysis

Statistical analyses were conducted using IBM SPSS Statistics (Version 27.0; IBM Corp, Armonk, NY). Propensity score matching (PSM) was performed at a 1:3 ratio between women with and without prior gestational diabetes mellitus (PGDM vs non-PGDM), with the following matching covariates: (1) maternal age at delivery (years), (2) interpregnancy interval (months), and (3) ethnicity. Continuous variables were presented as median (interquartile range [IQR]) and compared using the Mann-Whitney U test. Categorical variables were expressed as frequencies (percentages) and analyzed by χ² test with Yates’s correction or Fisher’s exact test when appropriate (expected cell frequency <5). Binary logistic regression was employed to evaluate the association between prior GDM exposure and adverse pregnancy outcomes, utilizing an entry criterion of P<0.1 for variable selection and considering P<0.05 (two-tailed) as statistically significant.

## 3 Results

### 3.1 Characteristics of PGDM and non-PGDM groups

A total of 3,109 participants meeting the inclusion criteria were initially identified, of whom 332 (10.68%) had a history of PGDM. Prior to PSM, significant intergroup differences were observed in maternal age, interpregnancy interval (months), length of hospital stay, and gestational age at delivery (all P < 0.05). After 1:3 PSM matching on maternal age, interpregnancy interval, and ethnicity, these baseline characteristics were well-balanced between the groups (all P > 0.05), as detailed in **Table 1**.

**Table 1:** Comparative results of the PGDM and Non-PGDM groups on baseline characteristics.

Characteristics	Non-PGDM (N = 994)	PGDM (N = 332)	Z/X^2^	P
Age	31.00 (29.00, 34.00)	31.00 (29.00, 34.00)	-0.075	0.940
Interpregnancy interval in months	34.00 (24.00, 50.00)	36.50 (24.25, 49.75)	-0.520	0.603
Ethnicity (Minority) n,(%)	33/961(3.3)	12/320 (3.6)	0.066	0.797
Office, n(%)			3.453	0.327
worker	54 (5.4)	10 (3.0)		
staff	34 (3.4)	11 (3.3)		
self-employed	41 (4.1)	12 (3.6)		
other	865 (87.0)	299 (90.1)		
Blood type, n(%)			1.238	0.744
A	273 (27.5)	94 (28.3)		
AB	61 (6.1)	25 (7.5)		
B	276 (27.8)	85 (25.6)		
O	384 (38.6)	128 (38.6)		
Number of pregnancy n (%)			2.239	0.327
2	525 (52.8)	160 (48.2)		
3	277 (27.9)	104 (31.3)		
≥4	192 (19.3)	68 (20.5)		
Number of delivery n (%)			1.673	0.433
2	793 (79.8)	266 (80.1)		
3	170 (17.1)	60 (18.1)		
≥4	31 (3.1)	6 (1.8)		
History of miscarriage, n (%)	372/622 (37.4)	143/189 (43.1)	3.342	0.068

The P values<0.1 are written in bold text.

### 3.2 Pregnancy outcomes in the PGDM group and non-PGDM group

Compared with the non-PGDM group, the PGDM group demonstrated a significantly higher incidence of cesarean section (32.8% vs. 26.9%, p=0.037), gestational diabetes (50.0% vs. 11.8%, p<0.001), and hypertensive disorders of pregnancy (3.0% vs. 1.2%, p=0.026). Notably, although the median gestational age at delivery was 39.0 weeks in both cohorts, the distribution differed significantly (p<0.001), suggesting a tendency toward earlier delivery among PGDM patients. Conversely, the incidence of postpartum anemia was lower in the PGDM group (16.9% vs. 23.1%, p=0.016). In contrast, no statistically significant differences were observed in other maternal complications—including preterm birth, premature rupture of membranes, eclampsia, meconium-stained amniotic fluid, antepartum or postpartum hemorrhage, thyroid dysfunction, or intra-amniotic infection—all showing p>0.05. Similarly, neonatal outcomes—such as sex distribution, birth weight, NICU admission rate, and birth weight-for-gestational-age categories—did not differ significantly between the two groups (all p>0.05), as detailed in **Table 2**.

**Table 2:** Comparison of pregnancy outcomes, obstetric outcomes, and neonatal outcomes between the PGDM group and the non-PGDM group.

Variable	Non-PGDM (N = 994)	PGDM (N = 332)	Z/X^2^	P
Cesarean section	267/727 (26.9)	109/223 (32.8)	4.366	0.037
Gestational diabetes	118/376 (11.9)	166/166 (50.0)	214.975	<0.001
Premature	31/963 (3.1)	13/319 (3.9)	0.493	0.483
Premature Rupture of Membranes	134/860 (13.5)	49/283 (14.8)	0.342	0.559
Eclampsia	29/965 (2.9)	13/319 (3.9)	0.808	0.369
Gestational hypertensive disorders	12/982 (1.2)	10/322 (3.0)	4.969	0.026
Meconium-Stained Amniotic Fluid	7/987 (0.7)	1/331 (0.3)	0.170	0.681
Gestational Anemia	222/772 (22.3)	72/260 (21.7)	0.060	0.806
Postpartum Anemia	230/764 (23.1)	56/276 (16.9)	5.786	0.016
Postpartum Hemorrhage	14/980 (1.4)	3/329 (0.9)	0.182	0.670
Antepartum Hemorrhage	3/991 (0.3)	2/330 (0.6)	0.066	0.797
Hypothyroidism in Pregnancy	60/934 (6.0)	24/308 (7.2)	0.597	0.440
Hyperthyroidism in Pregnancy	13/981 (1.3)	4/328 (1.2)	0.021	0.885
Infection of the amniotic sac and fetal membranes	34/960 (3.4)	13/319 (3.9)	0.178	0.673
Pregnancy with obesity	7/987 (0.7)	7/325 (2.1)	3.450	0.063
Days			5.456	0.065
<3 days	345 (34.7)	103 (31.0)		
3–5 days	552 (55.5)	182 (54.8)		
>5 days	97 (9.8)	47 (14.2)		
Weeks	39.00 (38.00, 40.00)	39.00 (38.00, 39.00)	-3.631	<0.001
Newborn outcomes				
sex, n(%)			0.063	0.801
Male	564 (56.7)	191 (57.5)		
Female	430 (43.3)	141 (42.5)		
Birth weight	3275.00 (3000.00, 3550.00)	3300.00 (3050.00, 3550.00)	-1.285	0.199
NICU, n(%)	84/910 (8.45)	35/297 (10.5)	1.333	0.248
BW-for-GA, n (%)			2.984	0.225
SGA	74 (7.4)	16 (4.8)		
AGA	847 (85.2)	288 (86.7)		
LGA	73 (7.3)	28 (8.4)		

The P values<0.1 are written in bold text.

### 3.3 Multivariate regression analysis

Following multivariable adjustment for potential confounders, a history of PGDM remained significantly associated with an elevated risk of recurrent gestational diabetes (OR = 7.320, 95% CI: 5.473–9.791, p < 0.001). It was also independently linked to a higher risk of gestational hypertension (OR = 2.924, 95% CI: 1.154–7.405, p = 0.024). Furthermore, PGDM history was correlated with a reduction in gestational length (per week) (OR = 0.881, 95% CI: 0.792–0.981, p = 0.021). Conversely, it was associated with a lower incidence of clinically significant postpartum anemia (OR = 0.694, 95% CI: 0.488–0.986, p = 0.042), as detailed in **Table 3**.

**Table 3:** The impact of a history of gestational diabetes on adverse pregnancy outcomes.

Variable	B	SE	Wald	p	OR	95% CI for OR
Weeks	-0.126	0.055	5.337	0.021	0.881	0.792-0.981
Gestational diabetes mellitus	1.991	0.148	179.996	<0.001	7.320	5.473-9.791
Gestational hypertensive disorders	1.073	0.474	5.119	0.024	2.924	1.154-7.405
Postpartum Anemia	-0.366	0.180	4.148	0.042	0.694	0.488-0.986
Constant	3.291	2.121	2.406	0.121	26.858	

P values<0.05 are written in bold text.

## 4 Discussion

This retrospective cohort study employed propensity score matching to systematically balance key confounding factors such as interpregnancy interval, maternal age (18), and ethnicity, providing more robust evidence that a history of gestational diabetes mellitus (GDM) constitutes an independent risk factor for adverse pregnancy outcomes in subsequent pregnancies.

The markedly elevated risk of GDM recurrence stands out as a principal finding. In this cohort, 50% of women with prior GDM developed GDM again in their subsequent pregnancy. Multivariate regression analysis revealed that a history of GDM is a strong independent predictor of recurrence (OR = 7.32), a finding consistent with previous studies indicating that PGDM substantially increases recurrence risk (7, 9, 19). From a pathophysiological perspective, the substantial increase in recurrence risk is closely associated with persistent insulin resistance and beta-cell dysfunction (18, 19). This suggests that PGDM may represent a persistent subclinical metabolic disorder, with pregnancy acting as a metabolic stressor that readily triggers its manifestation.

Beyond its impact on glucose metabolism, a history of GDM also independently increased the risk of gestational hypertensive disorders. This association may reveal a shared, deeper pathophysiological basis beyond simple glucose metabolism abnormalities. In addition to known metabolic factors such as insulin resistance, vascular endothelial dysfunction may serve as a key link connecting the two conditions. Pre-existing endothelial vulnerability in women with prior GDM may contribute to abnormal placental development (e.g., inadequate spiral artery remodeling), leading to reduced placental perfusion and upregulation of anti-angiogenic factors such as soluble fms-like tyrosine kinase 1 (sFlt-1), ultimately manifesting as gestational hypertension (20–22). Conversely, the exacerbated endothelial injury and systemic inflammatory (23) response caused by gestational hypertensive disorders may further worsen insulin resistance, thereby creating a vicious cycle.

Related to these metabolic and vascular complications, we observed a shift toward earlier delivery among women with prior GDM. Research indicates that women with prior GDM deliver at an earlier gestational age distribution (IQR: 38–39 vs. 38–40 weeks). This pattern is linked to planned early delivery for PGDM-related complications such as gestational hypertension. Beyond clinical management, the PGDM-associated metabolic milieu—characterized by hyperglycemia and insulin resistance—has been suggested to promote advanced glycation end-product accumulation and oxidative stress, which may theoretically affect uterine contractility (24), cervical ripening (25), and membrane integrity (26). Although the present study did not directly measure biomarkers to validate these pathways, the proposed mechanisms, grounded in existing literature, offer a plausible biological basis for earlier delivery. Taken together, the reduced gestational age observed in women with PGDM is likely multifactorial, involving both iatrogenic and pathophysiological determinants.

In contrast to these elevated risks, the incidence of postpartum hemorrhage did not differ significantly between the PGDM and non-PGDM groups; however, the rate of postpartum anemia was significantly lower in women with prior GDM. This distinct pattern may suggest that mechanisms beyond acute blood loss, particularly those involving iron metabolism and postpartum hematopoietic adaptation, could contribute to the reduced anemia incidence. Evidence consistently links elevated pre-pregnancy hemoglobin levels, indicative of greater iron stores, with an increased risk of GDM (27–29). Based on this evidence, it is hypothesized that women with a history of GDM may enter subsequent pregnancies with relatively higher baseline iron reserves. Given that postpartum anemia is determined not only by peripartum blood loss but also by the availability and mobilization of iron for erythropoiesis, a more favorable iron status could theoretically support more efficient hemoglobin regeneration after delivery, thereby attenuating the severity of anemia (30). Nevertheless, as this retrospective study did not include direct measurements of iron biomarkers (e.g., serum ferritin) or pre-pregnancy hemoglobin levels, the proposed mechanism remains speculative and warrants validation in future prospective studies with comprehensive iron status assessment.

In terms of neonatal outcomes, this study observed no statistically significant intergroup differences in key measures, including birth weight, BW-for-GA, and NICU admission rates. While this finding contrasts with some previous reports suggesting an increased risk of macrosomia associated with prior GDM (12, 31), it aligns with studies that found no independent association after rigorous adjustment for confounders (14). The reasons for this discrepancy may include the following: First, regarding the diagnostic criteria for GDM, although Guo et al.’s study adopted the IADPSG criteria, their blood glucose control targets were fasting glucose 3.3–5.3 mmol/L, 1-hour postprandial glucose <7.8 mmol/L, and 2-hour postprandial glucose <6.7 mmol/L, which differ from the ADA diagnostic thresholds used in this study; Second, regarding the definition of outcome measures, Guo et al. used a birth weight of ≥4000 g as the diagnostic criterion for macrosomia, whereas this study defined large-for-gestational-age (LGA) infants based on the 90th percentile of the Chinese neonatal growth curve, indicating a difference in classification criteria; Third, regarding the adjustment for confounding factors, Guo et al. performed only between-group comparisons without adjusting for confounders; although Wang et al. used multivariate logistic regression to adjust for factors such as maternal age, interpregnancy interval, and ethnicity, this approach cannot balance covariate distributions as effectively as propensity score matching. Furthermore, these differences may also stem from advances in GDM management strategies—including standardized glucose control during pregnancy and subsequent early-pregnancy interventions—which may effectively reduce the risk of fetal overgrowth (32).

Nevertheless, several limitations of this study should be acknowledged. First, propensity score matching included only maternal age, interpregnancy interval, and ethnicity, but not other important confounders such as pre-pregnancy body mass index and gestational weight gain. Owing to the retrospective nature of data collection, these variables were either incompletely recorded or lacked standardization; their inclusion would have substantially reduced the sample size and may have introduced selection bias, making residual confounding difficult to exclude entirely. Second, this was a single-center retrospective study; the geographic and clinical characteristics of the study population may limit the generalizability of our findings, and external validation in different populations and regions is warranted. Third, although the overall sample size was adequate, some subgroup analyses were underpowered due to limited case numbers, which may affect the robustness of the corresponding results. Fourth, this study lacked mechanistic validation data. The biological explanations proposed in the discussion (e.g., the iron reserve hypothesis, vascular endothelial dysfunction) are inferred from prior literature and were not directly validated using biomarkers or experimental data; thus, these mechanistic interpretations remain speculative. Fifth, this study focused primarily on perinatal outcomes and did not evaluate the long-term effects of PGDM on offspring health (e.g., metabolic or neurodevelopmental outcomes); future prospective studies are needed to address these questions.

## 5 Conclusions

This study establishes a history of PGDM as an independent risk factor for adverse maternal outcomes in subsequent pregnancies. Women with PGDM exhibited a substantially elevated risk of recurrent gestational diabetes (OR = 7.320, 95% CI: 5.473–9.791), a significantly higher incidence of gestational hypertensive disorders (OR = 2.924, 95% CI: 1.154–7.405), increased cesarean delivery rates, and a reduction in mean gestational age at delivery (OR per week = 0.881, 95% CI: 0.792–0.981). Interestingly, PGDM was associated with a decreased risk of postpartum Anemia (OR = 0.694, 95% CI: 0.488–0.986), indicating a multifaceted clinical phenotype. These findings underscore the need for enhanced preconception counseling—including metabolic assessment and weight optimization—and intensified prenatal monitoring, such as early glucose screening and regular blood pressure surveillance, in this population; clinical management should employ individualized strategies to mitigate pregnancy-related risks, and notably, given the observed lower risk of postpartum anemia, routine iron supplementation may warrant individualized adjustment. Further research is needed to elucidate the long-term effects of PGDM on offspring health and to clarify the pathophysiological mechanisms underlying both its adverse and protective associations.

## Data Availability

The raw data supporting the conclusions of this article will be made available by the authors, without undue reservation.

## Glossary

PGDM: Prior Gestational Diabetes Mellitus
OR: Odds ratio
CI: Confidence interval
PSM: Propensity score-matched
IVF: *In vitro* fertilization
AGA: Appropriate for gestational age
SGA: Small for gestational age
LGA: Large for gestational age
GA: gestational age
BW-for-GA: Birth weight-for-gestational age
SD: Standard deviation
IQR: Interquartile range
